# Antioxidant Enzymes and Their Potential Use in Breast Cancer Treatment

**DOI:** 10.3390/ijms25115675

**Published:** 2024-05-23

**Authors:** María Magdalena Vilchis-Landeros, Héctor Vázquez-Meza, Melissa Vázquez-Carrada, Daniel Uribe-Ramírez, Deyamira Matuz-Mares

**Affiliations:** 1 Departamento de Bioquímica, Facultad de Medicina, Universidad Nacional Autónoma de México, Avenida Universidad 3000, Cd. Universitaria, Mexico City C.P. 04510, Mexico; vilchisl@unam.mx (M.M.V.-L.); hvazquez@bq.unam.mx (H.V.-M.); 2 Institute of Microbiology, Cluster of Excellence on Plant Sciences, Heinrich Heine University Düsseldorf, 40225 Düsseldorf, Germany; m.vazquez-carrada@hhu.de; 3 Departamento de Ingeniería Bioquímica, Escuela Nacional de Ciencias Biológicas, Instituto Politécnico Nacional, Av. Wilfrido Massieu 399, Nueva Industrial Vallejo, Gustavo A. Madero, Mexico City C.P. 07738, Mexico; daniel.uriberam@gmail.com

**Keywords:** antioxidant, breast cancer, superoxide dismutase, catalase, glutathione peroxidase, glutathione reductase, thioredoxin reductase, peroxiredoxin

## Abstract

According to the World Health Organization (WHO), breast cancer (BC) is the deadliest and the most common type of cancer worldwide in women. Several factors associated with BC exert their effects by modulating the state of stress. They can induce genetic mutations or alterations in cell growth, encouraging neoplastic development and the production of reactive oxygen species (ROS). ROS are able to activate many signal transduction pathways, producing an inflammatory environment that leads to the suppression of programmed cell death and the promotion of tumor proliferation, angiogenesis, and metastasis; these effects promote the development and progression of malignant neoplasms. However, cells have both non-enzymatic and enzymatic antioxidant systems that protect them by neutralizing the harmful effects of ROS. In this sense, antioxidant enzymes such as superoxide dismutase (SOD), catalase (CAT), glutathione peroxidase (GPx), glutathione reductase (GR), thioredoxin reductase (TrxR), and peroxiredoxin (Prx) protect the body from diseases caused by oxidative damage. In this review, we will discuss mechanisms through which some enzymatic antioxidants inhibit or promote carcinogenesis, as well as the new therapeutic proposals developed to complement traditional treatments.

## 1. Introduction

Cancer is one of the leading causes of death worldwide and is a crucial factor reducing life expectancy. In 2022, 19.96 million new cases and almost 10 million deaths caused by cancer were recorded [1]. Currently, the World Health Organization (WHO) recognizes breast cancer (BC) as the most common type of cancer on a global scale [2]. In 2022, according to the United Nations, BC was reported as the cancer with the highest incidence, with 2.29 million new cases, representing 11.5% of all cancer diagnoses worldwide [3,4].

In Latin America, BC is the most common and the deadliest cancer in women as well. In 2022, the recorded incidence was 114,900 diagnoses, coupled with a mortality rate of 13.2 per 100,000 inhabitants [3]. The high mortality in this region could be attributed to several factors, such as late-stage diagnosis, lack of access to specialized cancer hospitals, and limited health insurance coverage for high-cost medications [4].

In Mexico, during 2020, malignant tumors accounted for the death of 97,323 people. Among them, 8% were attributed to BC, causing the death of 7821 women and 58 men. The highest rate of female deaths caused by BC is recorded in the 60-year-old and older age group (49,08 per 100,000 women in this age group) [5]. BC is a multifactorial disease and depending on its etiology, it can be classified as sporadic or hereditary. Sporadic BC represents around 90% of cases, while hereditary BC corresponds to 10–15% of malignant breast tumors.

Several factors associated with BC exert their effects through the modulation of the oxidative stress state in cells [6,7]. This stress can cause genetic mutations or alterations in cell growth, favoring neoplastic development and generating reactive oxygen species (ROS) from endogenous sources (intracellular elements such as peroxisomes, mitochondria, cytochrome P450, and extracellular elements such as inflammatory cells) and exogenous sources (xenobiotics, metals, pathogens, drugs, and radiation) [8,9,10]. The generated ROS can activate many signal transduction pathways factors such as vascular endothelial growth factor (VEGF), hypoxia-inducible factor 1 alpha subunit (HIF1α), nuclear factor erythroid 2 (Nrf2), activator protein 1 (AP-1) and nuclear factor kappa light chain gene enhancer in activated B cells (NF-kB). These factors transcribe cell growth regulatory genes [11,12] and they can also trigger the activation of kinases such as mitogen-activated protein kinase (MAPK), extracellular-regulated kinase (Erk), c-jun NH-2 terminal kinase (JNK), and p38 MAPK, which are involved in cell migration and invasion [13]. These signaling cascades generate an inflammatory environment that leads to different events such as the suppression of programmed cell death, tumor proliferation, angiogenesis, and metastasis; all of these favor the development and progression of malignant neoplasms [14].

On the other hand, chemical carcinogens have been shown to abrogate cellular antioxidant systems and/or DNA repair systems [15]. Oxidative stress arises due to an imbalance between the production and the elimination of ROS and performs a fundamental role in the pathogenesis of various disorders and pathophysiological processes such as BC [6,16,17].

In this regard, it is known that the increment of the oxidative stress markers and the decrement of the antioxidant defense system are considered factors that correlate with the appearance and progression of BC [18]. The DNA of BC cells contains many base modifications and shows oxidation products, such as 8-hydroxydeoxyguanosine (8-OHdG), an element that appears to play a significant role in the progression of this disease [19]. Elevated levels of urinary 8-OHdG have been detected in women with BC, and this value becomes more significant in the late stage of cancer, a fact that suggests that ROS might play a role in early carcinogenesis [20]. ROS also appear to participate in the architecture distortion of the mammary epithelium, inducing fibroblast proliferation, epithelial hyperplasia, cellular atypia, and BC [21].

The function of both non-enzymatic and enzymatic antioxidant systems is to protect cells from oxidative stress and neutralize the damaging effects of ROS [11]. According to this, antioxidant enzymes such as superoxide dismutase (SOD), catalase (CAT), glutathione peroxidase (GPx), glutathione reductase (GR), thioredoxin reductase (TrxR), and peroxiredoxin (Prx) protect the body from diseases caused by oxidative damage. In this review, we will discuss various mechanisms through which some enzymatic antioxidants can either inhibit or promote carcinogenesis, as well as the new therapeutic proposals developed to complement traditional treatments.

## 2. Oxidative Stress

During cellular metabolism, many short- and long-lived ROS are generated: superoxide (O_2_^·−^), hydroxyl radical (OH^∙^), and hydrogen peroxide (H_2_O_2_). Depending on their concentration, location, and intracellular conditions, ROS can cause toxicity or act as signaling molecules [22]. This ROS dual role is supported by growing evidence that has shown they act within cells, either as second messengers in intracellular signaling cascades or by inducing and maintaining the oncogenic phenotype of cancer cells. On the other hand, ROS can also play a role in cellular senescence and apoptosis, functioning as antitumor species [23,24]. In cancer cells, ROS production is elevated due to an increased metabolic rate and relative hypoxia, resulting in the induction of genetic mutations and changes in transcriptional processes and ultimately, in the development of cancer [25]. Furthermore, cancer cells adapt to elevated ROS levels by activating antioxidant pathways, resulting in increased ROS scavenging [26].

## 3. Antioxidant Systems

**Figure 1:**
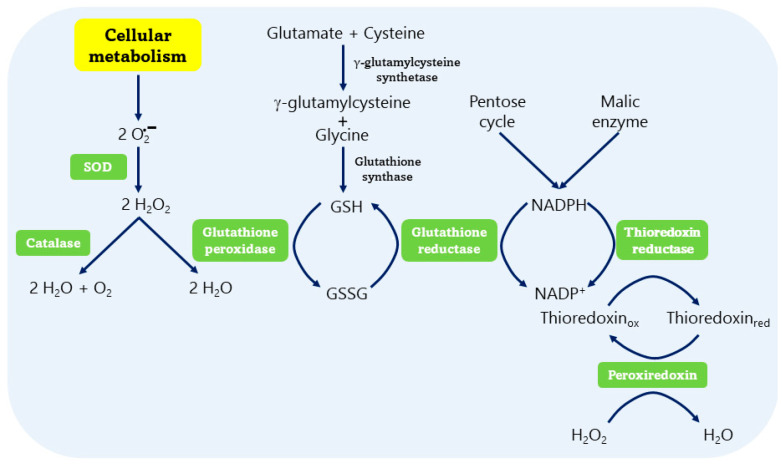
The antioxidant enzymatic system is made up of six enzymes: superoxide dismutase (SOD), catalase (CAT), glutathione peroxidase (GPx), glutathione reductase (GR), thioredoxin reductase (TrxR), and peroxiredoxin (Prx). SOD eliminates the 2 O_2_^·−^, generating two H_2_O_2_ molecules and CAT destroys them, producing H_2_O and O_2_. GPx, GR, TrxR, and Prx remove H_2_O_2_ by regulating the redox conditions of glutathione, thioredoxin, and NADPH.

The cellular levels of ROS are controlled by two well-established mechanisms: (i) non-enzymatic antioxidant systems, such as glutathione, thioredoxin, and vitamins like C and E; and (ii) enzymatic antioxidant systems (Figure 1). This battery of enzymes, involved in the control of ROS, includes SOD, CAT, GPx, GR, TrxR, and Prx [27,28].

### 3.1. Non-Enzymatic Antioxidant Systems

Non-enzymatic antioxidant barriers are a set of compounds that are obtained from the diet [29] and they work together with antioxidant enzymes [27]. These are also known as scavenger antioxidants since they counteract the beginning of the oxidation chain and interrupt its propagation reactions, thus neutralizing ROS by donating electrons to them [30,31]. Some non-enzymatic antioxidants are glutathione; thioredoxin; lipoic acid; bilirubin; ubiquinones; flavonoids; vitamins A, C, and E; and carotenoids [27,29]. The minerals selenium, copper, zinc and magnesium are part of the molecular structure of some of the antioxidant enzymes [32].

### 3.2. Enzymatic Antioxidant Systems

#### 3.2.1. Superoxide Dismutase (SOD)

The function of SOD is to convert O_2_^·−^ anions into H_2_O_2_ and oxygen (O_2_), while other enzymes, such as peroxidases and CAT, convert H_2_O_2_ into water [33]. SODs are the first line of defense against ROS-mediated injuries. The SOD family includes copper and zinc-dependent SOD (Cu/ZnSOD or SOD1), manganese-dependent SOD (MnSOD or SOD2), iron-dependent SOD, and extracellular superoxide dismutase (FeSOD or SOD3 or EcSOD) [34], and all of these play a crucial role in the removal of O_2_^·−^ [22]. SODs are involved in the regulation of oxidative stress, lipid metabolism, inflammation, and oxidation in cells. Furthermore, they can prevent lipid peroxidation, oxidation of low-density lipoproteins in macrophages, formation of lipid droplets, and adhesion of inflammatory cells to endothelial monolayers [35]. Additionally, it has been reported that there is a significant decrease in the activity of Cu/ZnSOD and MnSOD in cancer cells (Table 1) [35,36,37].

#### 3.2.2. Catalase (CAT)

CATs are enzymes that break down H_2_O_2_ into O_2_ and H_2_O [90]. They have a key role in the defense of cells against oxidative stress and their overexpression modulates the H_2_O_2_ levels and inflammation processes [91]. Catalase expression is altered in cancer cells, a fact that plays an important dichotomous role [40]. These enzymes are classified into three types: typical, peroxidases, and with manganese. The first two types of enzymes contain a heme group, while the third type contains a non-heme manganese group. Typical CATs are commonly isolated from aerobic organisms, including animals, plants, fungi, and bacteria. CAT-peroxidases have been isolated from fungi, eubacteria, and archaea, while non-heme CATs have been found exclusively in bacteria [92]. Human CAT belongs to the group of typical CATs [90] (Table 1).

In BC, blood samples have shown an increase of ROS production, a decreased CAT activity, as well as a low concentration of reduced glutathione (GSH). Those results support the hypothesis of oxidative stress in carcinogenesis [43]. In some studies, it has been observed that mammography density in benign tissue is influenced by markers of oxidative stress and the menopausal status of the patient [93]. Furthermore, CAT activity decreases with the progression of the pathology according to the Breast Imaging Reporting and Data System (BIRADS). Randecovic et al. in 2013 showed how the levels of CAT change in tumor tissue from patients with BC with respect to mammographic studies. Patients with BIRADS 5 had significantly lower levels of CAT activity compared to patients with BIRADS 4c, 4b, or 4a mammography. So, patients with BIRADS 4c had significantly lower CAT activity compared to patients with BIRADS 4a or 4b on mammography. Therefore, they concluded that the CAT activity in the BC decreased significantly with the increase in the BIRADS category [94]. This work is in agreement with other studies published for BC, which show a decrease in CAT activity associated with an aggressive phenotype [95].

Moreover, in acute myeloid leukemia it has been observed that cells from patients who remain in complete remission for longer periods have higher levels of CAT than patients who exhibit resistance to treatment; this suggests that the acquisition of cellular resistance to drugs is associated with higher levels of ROS [96]. In MCF-7 cancer cells (human adenocarcinoma cells), the overexpression of CAT affects their proliferation and migration, leading to a less aggressive phenotype and an altered response to chemotherapy, since they are more sensitive to paclitaxel, etoposide, and arsenic trioxide and more resistant to redox-based drugs [97]. Ruqayah et al. in 2020 showed decreased CAT activity and glutathione concentration levels in women patients with BC and they concluded that the biochemical changes in CAT and GSH levels can be viewed as biomarkers for the early detection of recurrent disease as well as for tracking the patients’ response to therapy during follow-up [98]. Zinczuk et al. found that the activity of SOD was significantly higher whereas the activity of CAT, GPx and GR was considerably lower in colorectal cancer (CRC) patients compared to the control group (*p* < 0.0001). They concluded that redox biomarkers can be potential diagnostic indicators of CRC advancement [99]. In addition, Glorieux et al. showed that CAT overexpression in mammary cancer cells leads to a less aggressive phenotype and an altered response to chemotherapy. The proliferation and migration capacities of MCF-7 cells were impaired by the overexpression of CAT as compared to parental cells. Regarding their sensitivity to anticancer treatments, they observed that cells overexpressing CAT were more sensitive to paclitaxel, etoposide and arsenic trioxide. However, no effect was observed in the cytotoxic response to ionizing radiation, 5-fluorouracil, cisplatin or doxorubicin. Finally, they observed that CAT overexpression protects cancer cells against the pro-oxidant combination of ascorbate and menadione, suggesting that changes in the expression of antioxidant enzymes could be a mechanism of resistance of cancer cells toward redox-based chemotherapeutic drugs [97].

These findings show that CAT can be a biomarker for the early detection, prognosis, and treatment of BC and cancer in general.

#### 3.2.3. Glutathione Peroxidase (GPx)

The main defense systems against the damage caused by H_2_O_2_ and lipid hydroperoxides are glutathione, thioredoxin, and CAT [100]. GPxs are a group of enzymes that use glutathione as a donor of reduced equivalents [101,102]. They convert H_2_O_2_ into H_2_O by oxidizing the glutathione (GSSG), which returns to its reduced form (GSH) through the action of the NADPH-dependent GR enzyme [102]. These enzymes are characterized by having selenium–cysteine in their active site and can reduce organic and inorganic hydroperoxides [100]. So far, based on their structural similarities, eight GPxs have been identified in mammalian cells [55,103,104] and their expression levels are altered in BC (Table 1).

GPx1 has been implicated in the development and prevention of numerous cardiovascular diseases and cancer [55,104]. In the case of cancer, it is known that in cell culture both GPx1 and GPx2 can prevent oxidative DNA mutations and counteract the production of proinflammatory mediators derived from cyclooxygenase (COX)/lipoxygenase (LOX), such as prostaglandins and leukotrienes. This capacity allows the prevention of carcinogenesis at least in the initial phase [105]. GPx4 regulates ferroptosis, which is an iron-mediated cell death mechanism. This pathway has been studied in several in vitro models and is under investigation as a potential therapeutic target for many types of cancer, including breast, ovarian, liver, and prostate cancer [59].

#### 3.2.4. Glutathione Reductase (GR)

GR is responsible for maintaining adequate concentrations of glutathione by converting GSSG into GSH; for that reason, its activity is relevant to maintaining the redox state of cells [106]. It has been determined that the levels of “activity and/or expression” of GR are increased in cancer cells, especially breast, lung, colorectal, and prostate cancer, thus affecting the concentration of GSSG in the cell and responses to treatment [106,107,108]. In addition, it has been found that in cell lines A-431, MCF-7, NCI-H226, and OVCAR-3, an increase in GR activity has been associated with resistance to radiotherapy treatments [108,109]. On the other hand, GR inhibition leads to an accumulation of GSSG, making cells more susceptible to ROS damage [110].

The use of coordinated therapies to inhibit BC growth has been proposed, such as chemotherapies and the use of inhibitors that affect the activity of antioxidant enzymes, such as GR [111]. An example of this is the inhibition of GR and TrxR enzymes, producing a decrease in GSH and Trx levels in BC (Table 1). This can be considered an effective strategy to combat tumor cells by disrupting their ability to eliminate ROS and cope with oxidative damage [112]. Studies that have observed the inhibition of cells indicate that their antitumor activity could be attributed to the inhibition of GR and TrxR activities [113].

#### 3.2.5. Thioredoxin Reductase (TrxR)

TrxR catalyzes the NADPH-dependent reduction of Trx [114,115]. By preserving protein thiols in a reduced state, Trx and TrxR help maintain a reduced cellular environment and keep transcription factors active [116]. These enzymes are a family of selenium-containing pyridine nucleotide-disulfide oxidoreductases [117]. There are three isoforms of TrxR (Table 1): TrxR1 located in the cytosol, TrxR2 located in the mitochondria, and TrxR3 or thioredoxin glutathione reductase, which is located mainly in the testes, where it participates in the maturation of the sperm [89,118].

TrxR1 is an oxidoreductase that has an active site with a dithiol disulfide, which in turn reduces oxidized cysteine residues in cellular proteins. This cytosolic enzyme is overexpressed in many types of human cancers, including BC, which is the reason for its usage as a biomarker [118,119]. Regarding TrxR2, it has been suggested that it has a protective role in cells during exogenous attacks such as radiation and certain anticancer drugs. It has been suggested that along with Prx, it regulates the release of cytochrome c, protecting cells from cancerous apoptotic processes [120].

#### 3.2.6. Peroxiredoxin (Prx)

Prxs are a family of peroxidases that are divided into six groups (Prx I–VI), and all of them can reduce H_2_O_2_ through Trx or other oxidative substrates [67]. They play a role as H_2_O_2_ sensors in cell signaling and differentiation systems, apoptosis, and redox homeostasis [73,121]. Human Prxs are expressed in different subcellular compartments [82] and the specific function of each isoform is related to their oligomeric and redox states [122]. Those oligomeric states could be influenced by ionic strength, pH, or protein concentration [123]. Moreover, they can be inhibited by tyrosine or threonine phosphorylation, and also by hyperoxidation [82]. These enzymes are also known as redox relays, yet they can transfer disulfide groups through proteins for redox signaling [124].

Prxs are overexpressed in many cancer tissues, suggesting that their proliferative effect could be related to the development or progression of the disease [67]. Mammalian tumors show high levels of Prx expression, indicating that antioxidant defenses provide a survival advantage for tumor development (Table 1). Consequently, Prx inhibitors are being explored as therapeutic agents in different cancer models and this enzyme is proposed as a potential biomarker in cancer due to the overexpression profiles found in malignant mammalian cells associated with survival [125].

## 4. Dichotomy of Some Antioxidant Enzymes

There is a “dichotomy” regarding the role of some antioxidant enzymes in cancer [126,127]. An example of this phenomenon is observed in the case of CAT, where a decrease in the activity of this enzyme is observed in BC; however, its activity has been positively correlated with advanced invasive and metastatic phenotypes in vivo in BC [94,127]. This enzyme is also frequently found to be decreased in acute myeloid leukemia (AML) and colorectal, pancreatic, and prostate cancers [40].

On the other hand, there is sufficient evidence from experimental and clinical studies regarding high levels and activity of SOD in tumor tissues [128]. In the most recent study on SOD dichotomy, researchers evaluated the levels of Mn-SOD, CuZn-SOD, and other antioxidant enzymes such as CAT and GPx in both tumors and adjacent non-tumor tissue from colorectal cancer patients. That study found that tumors exhibited higher levels of Mn-SOD. Notably, this enzyme’s level increased in stages II and III in comparison to stage I and the precancerous stage [129]. Moreover, some researchers claim that SOD2 undergoes a functional shift, transitioning from a tumor initiation suppressor to an actual tumor promoter, contributing to the progression toward more malignant phenotypes once the disease is established [130]. These observations are further supported by highlighting the importance of aerobic glycolysis in the survival of tumor cells [130]. Increased Mn-SOD suppresses mitochondrial oxidative phosphorylation and leads to the activation of MAPK where glucose is metabolized through glycolysis [128]. The change in the glycolytic phenotype of malignant cells is considered an adaptative advantage that reduces their dependence on mitochondrial respiration and allows them to proliferate and invade normal peritumoral tissue [131].

In humans, most Prxs show an increase in their expression levels during BC development [67,73]. Altered expression levels of CAT and Prx2 enzymes have been identified in nipple secretions [132], as well as the Prx2 and Prx6 in the serum of BC patients [133]. Furthermore, the detection of elevated levels of Prx1 in biopsies is associated with a better prognosis in estrogen receptor-positive BC [134]. Although its mechanism to protect cancer cells remains unclear, Prx is also known for participating in signaling pathways such as cell proliferation and death [121].

The functions of GPx change depending on the type of cancer and the type of study. GPx1 can reduce tumor growth, indicating its inhibitory effect on tumorigenesis [135]. GPx1 expression is decreased in BC [136], while in colorectal cancer, GPx2 expression is increased [137]. Conversely, in prostate intraepithelial neoplasia, its expression is decreased [138], which suggests that GPx2 shows diverse and complex functions in tumorigenesis. Furthermore, GPx3 is considered a tumor suppressing protein [139], and its expression is decreased in patients with Barrett’s esophagus [140], endometrial adenocarcinoma [141], and breast and prostate cancers [142]. GPx4 is also considered a tumor suppressor [135,139]. On the other hand, GPx7 has potential tumor suppressive effects in gastric and esophageal adenocarcinoma [143]. The roles of GPx5, GPx6, and GPx8 in tumorigenesis are still unclear [54].

Despite the large amount of information found in the literature on GR and TrxR, insufficient data have been found to support their possible dichotomous role in cancer.

## 5. Ferroptosis

**Figure 2:**
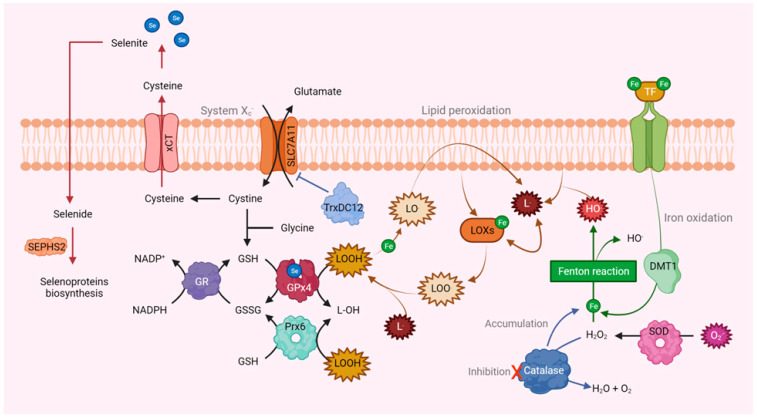
Relationship between antioxidant enzymes and ferroptosis. GPx4, Prx6, and GR act as negative regulators of iron-mediated death. GPx4 and Prx6 use GSH as a reducer and by inhibiting them it is possible to activate ferroptosis. Similarly, the concentration of these selenoproteins can be reduced by inhibition of selenophosphate synthetase 2 (SEPHS2), which is necessary for cancer cells to detoxify the selenide that enters through SLC7A11 and is useful in its biosynthesis. Furthermore, the GSH content can also decrease due to the activation of SOD and the modulation of the cystine-glutamate antiporter (SLC7A11) by TrdxDC12. Also, the inhibition of CAT causes the accumulation of Fe^2+^, favoring the Fenton reaction and, in turn, ferroptosis.

In the treatment of BC, resistance to apoptosis has been observed. Consequently, new procedures or therapies aimed at inhibiting its progression have been sought [59]. In this sense, ferroptosis, a type of cell death that is characterized by an excess of lipid peroxidation induced by ROS, might represent an effective treatment against aggressive tumors resistant to traditional drugs [54,59,144]. In BC, the modulators associated with ferroptosis are glutathione, GPx4, Prxs, iron, Nrf2, SOD, lipoxygenase, and coenzyme Q (Figure 2) [145].

In the case of GPx4, it has been shown that its inactivation results in an effective eradication of cancer cells by inducing cell death through ferroptosis [59,146]. As a result of this discovery, several pharmacological therapies have been developed to activate ferroptotic cell death by targeting GPx4. These therapies act on the biosynthesis, activity, or degradation of this enzyme [147,148,149].

Regarding CAT, it has been observed that thiosemicarbazone (BT-Br), a benzaldehyde derivative and an effective inhibitor of this enzyme, increases ROS levels in DU145 castration-resistant prostate cancer (CRPC) cells. BT-Br can induce endoplasmic reticulum stress and subsequent autophagy in these cells, causing the degradation of ferritin heavy peptide 1 (FTH1) and the accumulation of Fe^2+^. Consequently, these effects promote the Fenton reaction and ^•^OH is produced, inducing ferroptosis in DU145 cells and eventually reducing CRPC tumors. Overall, CAT inhibition has the potential to be a novel strategy to induce ferroptosis of cancer cells through the dual regulation of ROS levels and iron ions [150].

Moreover, GR is inversely related to ferroptosis because when it is overexpressed in cancer cells it regulates GSH homeostasis, rendering the cells insensitive to this process [151]. Concerning Prxs, Prx6 has been described as a negative regulator of cell death via ferroptosis, and it is suggested that through its Ca^2+^-independent phospholipase A2 inhibitory activity it eliminates the fatty acid hydroperoxide (LOOH), which protects cells against iron death. Therefore, its deletion results in a potential target for cancer therapy by inducing ferroptosis [152].

Regarding the role of the Trx/TrxR system in the regeneration of GSH, which protects against ferroptosis, TrxR should participate along with GPx4 in antiferroptotic activity. However, this is not the case, as the loss of TrxR1 protects pancreatic cancer cells from ferroptosis by inhibiting GPx4. The authors found that the loss of TrxR generates an increase in GPx4 protein levels by increasing the cellular pool of selenocysteine. Selenocysteine is an amino acid found in low intracellular concentrations, yet it is essential for the structure and function of both polypeptides. Hence, the disappearance of one induces the biogenesis of the second [153]. Certain drugs have been created to interfere in the redox homeostasis through the inhibition of TrxR and by inducing the death process through ferroptosis, which allows tumor cells to be sensitized to radiotherapy treatments [154].

However, despite how promising the induction of ferroptosis seems to be in the treatment of BC, further tests and a thorough examination of its safety and adverse effects in vivo must be carried out since preliminary studies have not reached clinical trials in human cancer therapy. Furthermore, it is necessary to consider that inhibiting CAT, GPx4, Prxs, TrxR, and other antioxidant enzymes can cause adverse reactions in other organs and tissues, which hinders their widespread application in human cancer. For these reasons, the development of inhibitors and more effective and specific targeting methods are needed to achieve optimal effects in the selective elimination of resistant human cancers, as well as the reduction of the potential adverse effects associated with ferroptosis.

## 6. Nanotechnology Applied to the Clinic

**Figure 3:**
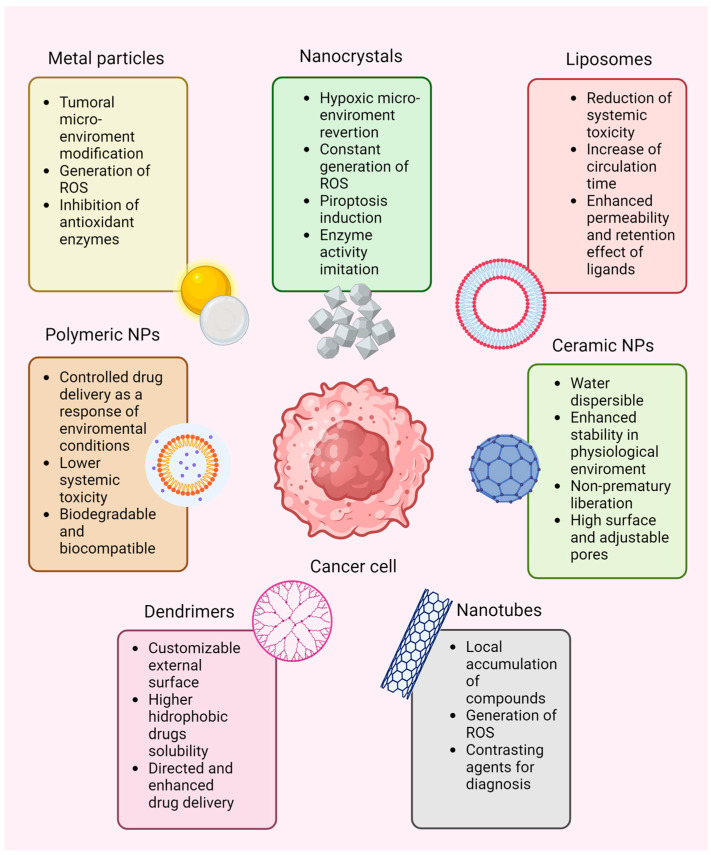
Characteristics of some nanoparticles used in nanomedicine in the treatment of BC.

Nanotechnology has emerged as a valuable tool that can be used to address the problem of cancer. Compared to conventional medicine, nanoparticles (NPs) offer advantages such as biocompatibility and affinity, lower immunogenicity [155], higher half-time life of attached drugs in blood circulation, specific accumulation in cancer tissues for a longer and more accurate effect [156], metastasis prevention [157], cell cycle arrest [158], and metabolic stress due to the low glycolysis level. Additionally, leveraging these characteristics, NPs can function as imaging agents for tumor monitoring and as a way to remodel the microenvironment with minimal to no toxic side effects on the patients [159]. Figure 3 and Table 2 show some of the compounds that have been employed in the treatment of BC using nanotechnology and antioxidant enzymes.

Within the NPs, nanozymes (artificial enzymes) have been developed to combine the characteristics of nanomaterials and enzymes. Natural enzymes often entail high production costs, susceptibility to denaturation and inactivation, and low yields, which make their application in different procedures difficult. However, nanozymes have high catalytic activities and share properties with nanomaterials, which could improve natural enzyme deficiencies. Nanozymes have been developed for CAT, SOD, oxidase, and peroxidase [182].

On the other hand, many drugs used in BC treatment increase the ROS levels to induce cell damage but the cytoprotective effect of antioxidant enzymes allows the cells to adapt to the new conditions and then survive. In this case, the metal particles coupled in NPs can directly inhibit antioxidant enzymes, and the carried chemical compounds’ action is effectively enhanced [179].

For example, the use of manganese dioxide (MnO_2_) and gold–platinum (Au-Pt) NPs helps to modify the tumor immunosuppressor microenvironment because they act as generators of radioactive nano-oxygen (nanobubbles composed of gas such as oxygen), improving the infiltration of cytotoxic T cells, affecting the cancer cells’ metabolism and reducing their proliferation by downregulating the expression of hypoxia-inducible factor 1α (HIF-1α) and C-MYC [183], and consequently, tumor progression [184,185].

In addition to this, the use of LaFeO_3_ perovskite nanocrystals–enzymes, which present enzyme-mimicking activities, including oxidase, peroxidase, GPx, and CAT activities, reverse the hypoxic microenvironment and the depletion of endogenous glutathione and promote the continuous production of ROS and the process of pyroptosis, presenting an alternative in the treatment of BC [186].

Moreover, nanozymes can be coupled to carbon nanotubules; for instance, a peroxidase activity nanozyme comprising a 1D ferriporphyrin covalent organic framework coated on a carbon nanotube (COF-CNT) with a conductor hydrogel can be injected into tumor tissues to generate ROS. Its use in the treatment of 4T1 BC, in mice, demonstrated that carcinoma was significantly suppressed [187].

It is known that NPs diminish the adverse effects of common cancer therapies [188] but their self-side effects are not well-documented yet and some of them can cause toxicity via cellular function disruption and initiate cell degradation through autophagy (e.g., gold, titanium dioxide or zinc oxide particles) [156]. Therefore, NPs must fulfill specific characteristics for clinical usage: 1. They need to be made of biocompatible and nontoxic elements, 2. their size should be small (10–200 nm), 3. more than 50% of the chemical compound is required to be encapsulated within the particle, 4. NPs should be stable in the physiological environment to prevent sequestration or agglomeration in undesirable sites, 5. they must have a reasonable circulation time, 6. they should target specifically the selected tissue or cell type, 7. the release of the attached compounds needs to be biological or extrinsically controlled, and finally, 8. the clearance of the particle needs to be ensured to prevent cumulative or long-term effects [188]. The developed NPs do not meet most and certainly not all of these characteristics, but with the required studies (absorption, metabolism, excretion, etc.) their application would improve the future of BC treatments.

The use of various types of NPs (liposomes, polymeric compounds, micelles, dendrimers, carbon nanotubes, etc.) has achieved significant advances in BC treatment due to their advantages compared to conventional therapies, such as reduced pharmaceutical toxicity and overcoming the chemoresistance of chemotherapy [189]. However, there are still several problems that remain to be addressed, so it is necessary to develop safer and more efficient BC treatments based on nanotechnology combined with the classical biochemical and immunological tools that have shown good results.

Many drugs used in the treatment of BC increase ROS levels to induce cellular damage, but the cytoprotective effect of antioxidant enzymes allows cells to adapt to the new conditions and then survive. In this case, metal particles coupled in NPs can directly inhibit antioxidant enzymes, and the action of the transported chemical compounds is effectively enhanced [179].

BC can be classified into three main groups based on their molecular and histological differences: BC expressing hormone receptors [estrogen receptor (ER^+^) or progesterone receptor (PR^+^)], BC expressing human epidermal receptor 2 (HER2^+^), and triple negative breast cancer (TNBC) (ER^−^, PR^−^, HER2^−^) [190,191]. In addition, TNBC is divided into six categories: basal-like 1 (BL-1), basal-like 2 (BL-2), immunomodulatory (IM), mesenchymal (M), mesenchymal stem cell-like (MSL), and luminal androgen receptor (LAR) [190], which makes the treatment protocol more complex. Therapy must be based on the molecular and histological characteristics of the type of BC.

Different investigations have been conducted to determine the efficiency of NPs on different types of cancer; among them, the incorporation of hormonal agents into NPs coated with polyethylene glycol to enter BC cells are working [192]. Furthermore, Gao et al. incorporated the TYKERB, lapatinib, into lipid NPs to improve their blood circulation time and bioavailability [193,194]. Other studies have indicated that BC cells could easily take up lipid NPs, thereby reducing tumor growth in mice. One of the key limitations of the clinical use of RNA is its rapid intravascular degradation. According to the findings of Wang et al. [194] in this regard, RNA loading onto lipid NPs caused an increase in intracellular siRNA uptake by MDA-MB-468 TNBC cells in vitro. In photothermal ablation (PTA), NPs (especially metallic NPs such as gold and silver) could produce heat at temperatures above 50 °C in BC tissues by absorbing and transforming energy from near-infrared waves, which results in the disruption of the cancerous tumors through necrosis.

Finally, the continuous development of NPs technology allows them to act on different types of BC. This generates greater efficiency in the death of cancer cells, which considerably reduces the toxic effects of traditional treatments (e.g., chemotherapy). In addition, the pharmacological effect becomes more effective and long-lasting, so healthy tissues are not affected. Hence, future research in this regard is expected to be directed toward the use of specific NPs at human clinical levels with minimal damage to surrounding healthy cells [156].

## 7. Conclusions

BC has a very high incidence worldwide, particularly in Latin America. Public health systems in this part of the world are seriously lagging in terms of prevention, a timely diagnosis and adequate guidance for patients with BC. This delay increases the mortality rate due to the poor availability of medications, long waiting lists for screening and clinical diagnosis, a low number of centers specialized in cancer care, a lack of trained personnel, centralization of health services, social inequality, and a deficiency of campaigns that encourage self-examination, among others. For these reasons, it is necessary to improve public health policies to strengthen research, development and the implementation of new oncological treatments, complementary to existing ones, to benefit a greater number of patients. Furthermore, it is necessary to implement new therapeutic targets, using antioxidant enzymes and biotechnological strategies such as ferroptosis and NPs, which will allow patients to be offered a more efficient treatment with fewer side effects, providing a better quality of life.

## Figures and Tables

**Table 1:** Antioxidant enzymes that participate in the regulation of breast cancer: location and function.

Enzyme	Type	Tissue Expression	Cellular Localization	Pathological Function	Therapeutical Use	Sample Type	References
Catalase	Typical	Highest enzyme activity in liver and erythrocytes, high activity in kidney and adipose tissue, intermediate in lung and pancreas, and very low in heart and brain	Peroxisomes	Dichotomous role: Protection from tumor formation and progression; however, it is also necessary for tumor progression and metastasis. It is frequently decreased in breast tumors and blood of patients with BC and BC cell cultures	Increasing CAT levels in breast tumors decreases hypoxia and attenuates the tumoral microenvironment immunosuppression condition through tumor-associated macrophages reprogramming from M2 (pro-tumoral) to M1 (anti-tumoral) due to increased O_2_ in the tumor, reversing hypoxia-induced chemotherapy resistance	Serum and tissue samples from BC patients and human BC MCF-7 cell line	[38,39,40,41,42,43,44]
SOD1	CuZn-SOD	Pons, substantia nigra pars compacta, dorsal root ganglion, lateral nuclear group of the thalamus	Cytosol, nucleus, and mitochondria	Reduced expression and activity, generating an increase in oxidative stress within the cell	In BC cells, the decrease in NAD-dependent deacetylase sirtuin-3 (SIRT3) expression can be counteracted by an upregulation of SOD1. As a result, the total level of ROS in the mitochondria is maintained within a window compatible with cell survival. In addition, it was reported that, using a panel of mammary cell lines, SOD1 is overexpressed and SIRT3 is decreased	MCF-10A, MCF-7, MDA-MB-231, and MDA-MB-157	[45,46]
SOD2	Mn-SOD	Lungs, placenta, kidney, pancreas and uterus, cartilage, skeletal muscle, brain, and eye	Mitochondrial matrix	Reduced expression and activity, generating an increase in oxidative stress within the cell and mitochondria	Almost all tumors have reduced Mn-SOD activity. Extensive epidemiological studies have mainly focused on the Ala16Val dimorphism of Mn-SOD as a risk factor for BC. Ambrosone and his colleagues were the first to report Ala16-MnSOD as a risk factor for BC	Data were collected in a case–control study of diet and BC in western New York from 1986 to 1991. Caucasian women with incident, primary, histologically confirmed BC were frequency-matched by age and county of residence to community controls. Blood specimens were collected and processed from a subset of participants in the study (266 cases and 295 controls)	[37,47,48]
SOD3	Ec-SOD	Cardiovascular endothelium, lungs, and placenta. Displays moderate activity within the kidney, pancreas, uterus, cartilage, skeletal muscle, adipose tissue, brain, and eye	Extracellular	Reduced activity, OH^·^ levels are increased through the Fenton and Harber–Weiss reactions. In addition, the oxidation of NO^•^ mediated by superoxide is increased, generating high concentrations of peroxynitrite (ONOO^−^)	Ec-SOD overexpression inhibited in vitro proliferation, clonogenic survival, and invasion of a triple-negative breast cancer cell (TNCB) line, in part by suppressing heparanase-mediated cleavage of cell surface proteoglycans and by reducing the bioavailability of VEGFA (vascular endothelial growth factor A). Ec-SOD overexpression also significantly inhibited tumor metastasis in both an experimental lung and a mouse model of spontaneous metastasis	Non-malignant, post-stasis human mammary epithelial cells extracted from reduction mammoplasty, human mammary epithelial cells (HMEC) immortalized, non-malignant breast epithelial MCF-10A cells, human mammary adenocarcinoma cell lines, MCF-7 cells, MDA-MB-231 cells, and MDA-MB-435 cells	[48,49,50,51]
GPx1	Selenium-dependent	Red blood cells, liver, lung, and kidney	Cytosol, nucleus, and mitochondria	Acts as a tumor promoter by regulating the proliferation, invasion, migration, apoptosis, immune response, and drug sensitivity of tumor cells	Enzyme glutamate dehydrogenase 1 (GDH1) controlling the intracellular levels of alpha-ketoglutarate (α-KG) and subsequent metabolite fumarate. Fumarate binds to and activates GPx1, leading to attenuated cancer, cell proliferation, and tumor growth (cell culture) (decreased GPx1 expression in tumorous breast tissue) (in human BC cell lines, GPx1 is downregulated)	BC cell line MDA-MB-231. Tissue samples from human patients aged 44–82 years with BC and MCF-7 human carcinoma cells. Human BC MCF-7 and MDA-MB-231 cell lines compared with healthy breast MCF-10A cells	[52,53,54,55]
GPx2	Selenium-dependent	Gastrointestinal tract, breast	Cytosol and nucleus	It is upregulated in a variety of tumor cells and is associated with tumor cell proliferation and a poor prognosis of patients. It causes vascular malfunction and hypoxia	Once a cell has been programmed to proliferate in an uncontrolled way, GPx2 supports the growth of cells by inhibiting apoptosis. GPx2 loss stimulates malignant progression due to reactive oxygen species/hypoxia inducible factor-α (HIF1α)/VEGFA signaling, causing poor perfusion and hypoxia (in human BC cell lines, GPx2 is upregulated)	MCF-7 and MDA-MB-231 cell lines compared with healthy breast MCF-10A cells	[7,52,54,56]
GPx3	Selenium-dependent	Kidney, lung, epididymis, breast, heart, and muscle	Plasma and mitochondria	Reduced expression can promote the proliferation, motility, and invasion of melanoma cells	GPx3 directly targets the ERα gene in white adipose tissue, for which it was proposed as an important mediator of the estrogen effects in association with fat mass. Considering the link between visceral fat and BC initiation and progression, it is reasonable to observe an overexpression of GPx3 in BC cells (in human BC cell lines, GPx2 is upregulated)	MCF-7 and MDA-MB-231 cell lines compared with healthy breast MCF-10A cells	[7,57]
GPx4	Selenium-dependent	Thyroid gland, bronchus, duodenum, lung, breast, heart, and muscle	Nucleus, cytosol, and mitochondria	Increased expression may promote the malignant progression of BC	It is an inducer of ferroptosis and apoptosis through ubiquitination of GPx4 (GPx4 is downregulated, with reduced expression in several cell lines, including human BC)	MCF-7 and MDA-MB-231 cell lines compared with healthy breast MCF-10A cells	[7,54,58,59]
GPx5	Non-Selenium-dependent	Epididymis	Extracellular	Downregulated	In human BC cell lines, GPx5 is downregulated	MCF-7 and MDA-MB-231 cell lines compared with healthy breast MCF-10A cells	[7]
GPx6	Selenium-dependent	Olfactory epithelium	Epithelium	No data	No data	No data	[52]
GPx7	Non-Selenium-dependent	Preadipocytes	Lumen of the endoplasmic reticulum.	Downregulated	In human breast cancer cell lines, GPx7 is downregulated	MCF-7 and MDA-MB2-31 cell lines compared with healthy breast MCF-10A cells	[7,52]
GPx8	Non-Selenium-dependent	Lung	Transmembrane of the endoplasmic reticulum	Expression is upregulated	In human BC cell lines, GPx8 is upregulated. If GPx8 is suppressed in these cells, they express a non-functional IL-6 receptor, which does not interact with IL-6. This altered binding hinders the activation of the JAK/STAT3 signaling pathway, thus inhibiting the transition of cancer cells to an aggressive phenotype	Human breast cancer cell line MDA-MB-231	[54,60]
GR	Selenoprotein	Pylorus, islet of Langerhans, epithelium of nasopharynx	Mitochondria, nucleus, and cytoplasm	Protects cancer cells against increased oxidative stress and provides a survival advantage	Increased GR activity in tumor cells and in the blood of BC patients. Therefore, inhibition of glutathione reductase in BC cells causes increased oxidative stress in the cell, which stops the growth of the cancer cell	The studies have been carried out on tissue samples from human patients with BC, aged 20 to 65 years; some were taken from ductal carcinoma; in the case of human BC cell lines, T-47D and MCF-7, D492 have been used	[61,62,63,64]
PrxI	2-Cysteine peroxidase	Thyroid gland, nasal cavity epithelium, olfactory segment of nasal mucosa, palpebral conjunctiva	Cytoplasm, melanosome, nucleus	Overexpressed in BC tissue. Correlated with shortened patient survival	Inhibition of PrxI gene upregulation may cause disadvantage to the survival and proliferation of tumor cells	Tissue from BC (type I to IV stage) patients	[65,66,67,68,69]
PrxII	2-Cysteine peroxidase	Thalamus, trabecular bone tissue, substantia nigra pars compacta, substantia nigra pars reticulata	Cytoplasm, nucleus	Overexpressed in BC tissue. Induces carcinogenic changes, maintains cancer stem cells phenotype and stemness properties	Inhibition of PrxII with siRNA partially reverses the radioresistant phenotype in radiation-resistant BC cells	Tissue from BC (type I to IV stage) patients	[67,69,70]
PrxIII	2-Cysteine peroxidase	Adrenal tissue, adrenal gland cortex, heart right ventricle, biceps brachii	Mitochondrion, cytoplasm, early endosome	Overexpressed in BC tissue. Related to tumorigenesis	Potential proliferation marker. Related to a better prognosis	Human BC MCF-7 and MDA-MB-231 cell lines. Tissue from BC patients	[68,69,71,72,73]
PrxIV	2-Cysteine peroxidase	Pancreas, tibia, adrenal tissue	Cytoplasm, endoplasmic reticulum	Overexpressed in progesterone receptor positive cases. Promoted migration and invasion of cancer cells	Promising therapeutic target for inflammatory diseases and cancer. Related to a better prognosis	Tissue from BC patients	[73,74,75,76]
PrxV	2-Cysteine peroxidase	Bronchial epithelial cell, epithelium of nasopharynx, palpebral conjunctiva, fallopian tube (uterine tube)	Mitochondria, cytoplasm, peroxisome matrix	Overexpression of PrxV gene is correlated with a larger tumor size, positive lymph node status, and shorter survival. Deficiency induced M2 macrophage polarization	PrxV is a putative therapeutic target and clinical strategy in breast, bladder, lung, cervical, ovarian, prostate, esophageal, and hepatocellular tumors	Human BC MCF-7 cell line	[68,73,77,78,79,80]
PrxVI	1-Cysteine peroxidase (GSHs reductant)	Corpus epididymis, gastrocnemius, mucosa of stomach, amniotic fluid	Cytoplasm, lysosome, lamellar bodies, nucleus	Upregulated in progesterone receptor positive cases. Overexpression of PrxVI leads to a more invasive phenotype and metastatic potential in BC. Increased in most metastatic cell lines	Prx6 stable knockdown xenografts exhibited decreased tumor growth and metastasis	BC cell lines, xenograft tumor model in athymic mice	[81,82]
TrxR1	Selenocysteine-containing protein	Ovary, spleen, heart, liver, kidney, and pancreas	Cytoplasm	TrxR overexpression has been correlated with aggressive tumor growth, worse prognosis, and decreased patient survival.	Inhibition of TrxR causes malignant cells to become more susceptible to cytotoxicity, cytostasis, and cell death.	Human BC MDA-MB-435 S, MDA-MB-231, BT-549, and MCF-10A cell lines	[83,84,85,86]
TrxR2	Selenocysteine-containing protein	Pharyngeal and body wall muscles	Mitochondria	Overexpressed in cancer cells, conferring apoptosis resistance.	Increases the mitochondrial concentration of reactive oxygen species and shifts the thiol redox state toward a more oxidized condition	Human BC MCF-7 cell line	[87,88]
TrxR3	Selenocysteine-containing protein	Testis	No data	No data	No data	No data	[89]

Consult Supplementary Table S1 to check the characteristics of the mentioned cell lines.

**Table 2:** Nanotechnology targeting antioxidant enzymes in BC treatment.

Target Antioxidant Enzyme	Compound	Action Mode	References
SOD	PSE-PCF-NPs: Shell poly (lactic acid-co-glycolic) (PLGA)-NPs coated with folic acid (FA)-chitosan (PCF-NPs) loaded with *Peganum harmala* smoke extract (PSE)	The combination of chitosan and PGLA increases the bioavailability, toxicity, and release of the drug. In addition, the use of folic acid on the surface of the NPs is one of the most effective strategies to internalize into cancer cells through receptor-mediated endocytosis and the administration of anticancer agents. As a result, an increase in ROS and a decrease in the SOD enzyme was obtained in MCF-7 cells treated with PSE-PCF-NP.	[160]
	Fe_3_O_4_@mSiO_2_-DSF@PEI-FA, mMDPF: Disulfiram (DSF) loading, encapsulated folic acid (FA) conjugated polyethyleneimine magnetic mesoporous silica (Fe_3_O_4_@mSiO_2_) NPs	Disulfiram is a SOD inhibitor due to its affinity for sulfhydryl groups and the ability to bind to the copper and zinc of SOD. Inhibition of SOD can cause superoxide accumulation in cells inducing oxidative stress, apoptosis, and cell cycle arrest; it also reduces cancer cell proliferation, angiogenesis, tumor metastasis, and multidrug resistance. Fe_3_O_4_@mSiO_2_ is a drug carrier system based on magnetic mesoporous silica NPs and folic acid, and it is used to increase both its solubility in water and its specificity for cancer cells. Finally, the addition of Cu^2+^ increases the therapeutic effect of DSF in different types of cancer cells.	[161]
	PC + C_22_PEG_900_GlcNAc: Diethyldithiocarbamate (DETC), zinc phthalocyanine (ZnPc) loaded in liposomes	The encapsulation of photosensitizers with liposomes improves their therapeutic activity while preserving their photophysical properties, in addition to reducing their toxic effect. The principle of photodynamic therapy (PDT) involves the production of high levels of ROS photosensitizing molecules, which when exposed to visible light can kill nearby cells. ZnPc is a second-generation photosensitizer used in PDT to produce singlet oxygen, while DETC is a hydrophilic metal chelating agent and a known SOD inhibitor. Thus, the inhibition of SOD increases the ROS generated by PDT, causing an increase in the death of tumor cells.	[162]
CAT	PMO-Ce6@Catalase: Mesoporous organosilica (PMO) coupled with chlorine e6 (Ce6) and CAT	PMO-Ce6@Catalase is selectively absorbed and stored by tumor tissue. Then, after local irradiation with light of appropriate wavelength, the photosensitizer (Ce6@) is activated to produce a photosensitizing effect to generate high levels of ROS. CAT increases the concentration of oxygen around the cells and solves the problem of hypoxia in the tumor, in addition to enhancing the effects of Ce6@.	[163]
	CAT@PDL1-SSLs: CAT-loaded and PDL1 (programmed death-ligand 1) monoclonal antibody modified immunoliposomes	PDL1 monoclonal antibodies are used as immune checkpoint blockers (ICBs) to significantly improve the efficacy of tumor immunotherapy by blocking the PD-1/PD-L1 inhibitory pathway. CAT helps the system overcome hypoxia, which is a limitation for PDL1. The results of this nanoparticle are activating and increasing the infiltration of CD8+ T cells at the tumor site and inhibiting tumor growth with low systemic toxicity.	[164]
	FA-L@MBDP@CAT: Lyso-targeted NIR photosensitizer (MBDP), CAT and doxorubicin (Dox) encapsulated within folic acid (FA) modified liposomes	Increased M1-MQ polarization; increased recruitment of CD8+ T cells. Photosensitizer (MBDP) has deep tissue penetration and high phototoxicity toward cancer cells. Doxorubicin has a good therapeutic effect on BC and metastatic tumors by inducing DNA damage and inhibiting the progression of topoisomerase II enzyme. For these liposomes, folate increases active targeting and prevents them from being recognized and phagocytosed by the reticuloendothelial system (RES) due to the existence of the PEG framework. The released CAT catalyzes overexpressed hydrogen peroxide to increase tumor oxygenation, providing sufficient oxygen for PDT and reversing the immunosuppressive TME by modulating immune cytokines to favor antitumor immunities, enhancing tumor inhibition in vivo.	[165]
	CAT@Pt(IV)-liposome: CAT-loaded cisplatin constructed liposome	CAT is encapsulated together with cisplatin (IV), forming a CAT@Pt(IV) liposome to improve cancer chemoradiotherapy. After loading into the liposomes, the CAT within the CAT@Pt(IV) liposome shows retained and well-protected enzymatic activity and is capable of triggering the breakdown of H_2_O_2_ produced by tumor cells, to produce additional oxygen for relieve hypoxia. As a result, CAT@Pt(IV) liposome treatment induces the highest level of DNA damage in cancer cells after X-ray irradiation.	[166]
	CAT-TCPP/FCS: Assembled FCS (fluorinated chitosan) with meso-tetra(4-carboxyphenyl) porphyrin (TCPP) conjugated CAT	These NPs exhibit greatly improved transmucosal adsorption and intratumoral penetration, due to their abilities to reversibly modulate transepithelial electrical resistance (TEER) and open tight junctions of the bladder epithelium. Such actions, together with the in situ O_2_ generation triggered by the CAT-catalyzed decomposition of the endogenous H_2_O_2_ of the tumor, would contribute to drastically improve the efficacy of sonodynamic therapy to destroy orthotopic bladder tumors.	[167]
	ZCM nanocapsule: CAT and methylene blue co-loaded into mesoporous of zeolite nanocarriers	Free CAT efficiently modulates tumor hypoxia and enhances intratumoral contrast through sustained decomposition of endogenous H_2_O_2_ and in situ production of O_2_ gas bubbles. Meanwhile, loading methylene blue into zeolite matrices prevents rapid leaching of photosensitizer in tumor tissue, achieving well-sustained release effect of photosensitizer. According to synchronous mechanisms, after near-infrared laser irradiation, local pancreatic cancer cells die completely, and no therapy-induced toxicity or recurrence is observed.	[168]
	PLGA-R837@CAT: CAT and R837 co-loaded in core/shell poli (lactic acid-co-glycolic) (PLGA) NPs platform	Reduced tumor metastasis; increased M1-MQ polarization; enhanced immunological cell death. The formed PLGA-R837@CAT nanoparticles can greatly improve the efficacy of radiotherapy by alleviating tumor hypoxia and modulating the immunosuppressive tumor microenvironment. Antigens with R837 will induce strong antitumor immune responses, which together with the blockade of the cytotoxic T lymphocyte-associated protein 4 (CTLA-4) checkpoint will be able to effectively inhibit tumor metastases through a strong abscopal effect (the reduction or disappearance of tumors in parts of the body that were not the direct target of local therapy, such as radiotherapy.	[169]
GPx4	FCSP@DOX MOF: Fe and Cu ions bridged by disulfide bonds with PEGylation (FCSP MOFs) loaded with doxorubicin (Dox)	FCSP@DOX MOFs are structures activated by the redox environment of the tumor, which causes the release of Fe^2+^/Cu^+^ ions to produce ROS through the Fenton reaction, triggering the depletion of GSH levels and the inhibition of GPx4, which causes an elevation of lipid peroxidation and the onset of ferroptosis. Additionally, better tumor therapeutic efficiency is achieved by loading DOX, since it can not only cause apoptosis, but also indirectly produces H_2_O_2_ to amplify the Fenton reaction, which allows the notable antitumor effect of FCSP@DOX MOFs.	[170]
	ChA CQDs: Carbon quantum dots (CQDs) prepared into nanozymes from chlorogenic acid (ChA)	CQDs have GSH oxidase-like activity by catalyzing the conversion of GSH to GSSG. Due to this, ChACQDs induce ferroptosis by promoting an unbalanced redox reaction due to the depletion of GSH and the inactivation of GPx4, with the consequent accumulation of ROS and lipid peroxidation.	[171]
	HMTBF: Metal-polyphenol network coated Prussian blue NPs	The HMTBF nanocomplex promotes ferroptosis/apoptosis synergism. During the intracellular degradation of this nanocomplex, the Fe^3+^/Fe^2+^ conversion mediated by tannic acid (TA) is favored, initiating the Fenton reaction and increasing the level of ROS, subsequent lipid peroxidation and, therefore, causing ferroptotic cell death. Furthermore, the degradation of HMTBF allows the release of the compound ML210, which inhibits the activity of GPx4 to activate the ferroptosis pathway.	[172]
	DBCO-RSL3-DHA: Dibenzocyclooctyne-modified disulfide-bridged nanoassemblies loaded with RSL3 and dihydroartemisinin	DBCO-RSL3-DHA nanoassemblies are loaded with the ferroptosis inducer RSL3 and the ferritinophagy initiator dihydroartemisinin (DHA). DHA induces ferritinophagy to release iron in the form of Fe^2+^. The cellular abundance of Fe^2+^ is the driving force of lipid peroxidation, together with the inhibition of GPx4 caused by RSL3, which triggers iron-dependent cell death (ferroptosis).	[173]
	Cu-TCPP(Fe): Metal-organic framework (MOF) incorporated with gold NPs and RSL3	Cu^2+^ ions immobilized on Cu-TCPP(Fe) nanosheets rapidly oxidize GSH to GSSG, potentially depleting GPx4 cofactors to inactivate its antiferroptotic functions. Furthermore, the nanosheet system can release the attached RSL3, which binds to the catalytic selenocysteine residue of GPx4. Overall, the multienzyme reactivity can simultaneously inhibit the GPx4 and ferroptosis suppressor protein 1 (FSP1) pathways that catalyze the recycling of coenzyme Q10 to ubiquinol, both ferroptosis-suppressing mechanisms.	[174]
	(RSL3@COF-Fc): Ferrocene-functionalized covalent organic framework loaded with RSL3	The RSL3@COF-Fc nanodrug carries a GPx4 inhibitor and RSL3. This nanodrug promotes in situ reactions similar to the Fenton reaction, triggering the production of hydroxyl radicals (·OH) by increasing the level of ROS in cells and irreversible covalent inhibition of GPx4, resulting in massive lipid peroxide accumulation, cellular damage, and ultimately, ferroptosis.	[175]
	Mn12-heparin: Manganese cluster NPs (Mn12) encapsulated with heparin	Manganese (Mn12) NPs encapsulated with heparin (Mn12-heparin) are a chemodynamic therapeutic agent that mediates the increase in ROS levels since manganese reacts with H_2_O_2_ to generate ·OH through a pathway similar to the reaction of Fenton. Increased ROS and depletion of endogenous GSH indirectly leads to GPx4 inhibition, consequently increasing the level of lipid peroxidation to cause ferroptosis.	[176]
	PFP@Fe/Cu-SS MOF: Phloroglucinol, iron (Fe^3+^), and copper (Cu^2+^) are the corresponding coordination metals. Perfluoropentane (PFP) was loaded into MOF	The high concentration of GSH present in tumor cells will accelerate the breakdown of the PFP@Fe/Cu-SS MOF nanocarrier structure, producing the release of Fe^2+^/Cu^2+^ ions that react with H_2_O_2_, producing ·OH through the Fenton reaction, causing the depletion of GSH levels and inhibition of GPx4. In turn, this causes the accumulation of intracellular lipid peroxides to eventually induce ferroptosis.	[177]
GR	HSpyN: pyrimidine-2 thiol	Phosphine-gold(I) thiolate complexes are promising anticancer agents for antiproliferative activities in vitro and in vivo. The ability of HSpyN to inhibit the proliferation of human BC cells, MCF-7, was evaluated by measuring cell death through the induction of apoptosis. In addition, this compound is a potent inhibitor of GR.	[178]
Prx1	Auf-Asc-Men: Auronofin (Auf) treatment in combination with ascorbate (Asc) and menadione (Men)	Prx1 can protect TNBC cells from the effects of prooxidant compounds, while Asc and Men treatment increases H_2_O_2_ levels. When Auf is added, Prx1 is inhibited, so the effects of H_2_O_2_ cause rapid toxicity, irreversible cell damage, and as a consequence, cell death instead of adaptation or survival.	[179]
TrxR	Auf-MI-463: Auronofin plus MI-463	Menin-MLL inhibitors have been shown to be effective against BC. MI-463 unexpectedly induced ferroptotic cell death. In addition, heme oxygenase-1 (HO-1) was inhibited, which increased the effect of MI-463 plus Auf.	[180]
	Auf-anti-PD-L1: Auronofin plus anti-PD-L1 antibody	Auf treatment acts as a TrxR inhibitor, causing specific cell death and affects cell growth. Auf increased tumor infiltration of CD8 + Ve T cells in vivo and amplified the expression of the immune checkpoint PD-L1 in an ERK1/2-MYC-dependent manner. Furthermore, the combination of Auf with anti-PD-L1 antibody synergistically impaired the growth of 4T1.2 TNBC primary tumors.	[64]
	Auf-Vitamin C: Auronofin plus vitamin C	Auf simultaneously targeted the antioxidant systems thioredoxin and glutathione, causing cell death. AUF/VC combinations exerted synergistic H_2_O_2_-mediated cytotoxicity on BC cell lines.	[181]

## Supplementary Materials

The following supporting information can be downloaded at: https://www.mdpi.com/article/10.3390/ijms25115675/s1. References [195,196] are mentioned in Supplementary Materials.
