# Supplementary material for: Antioxidant Enzymes and Their Potential Use in Breast Cancer Treatment
Source: Int J Mol Sci. 2024 May 23;25(11):5675. doi: 10.3390/ijms25115675 (PMC11171593; doi:10.3390/ijms25115675)
Supplement: Supplementary file 1 [file ijms-25-05675-s001.zip › ijms-2992295-supplementary.pdf]

Table S1. Mentioned human cell lines in the review.

Name	Cell type	Morphology	Tissue	Disease	References
**A-431**	Epithelial cell	Epithelial	Skin; Epidermis	Epidermoid Carcinoma	[1]
**BT-549**	Epithelial cell	Epithelial	Breast; Mammary gland	Carcinoma; Ductal	[1]
**D492**	Epithelial cell	Epithelial	Breast; Mammary gland	Non-applicable	[2]
**DU145**	Epithelial cell	Epithelial	Prostate	Carcinoma	[1]
**MCF-10A**	Epithelial cell	Epithelial	Breast; Mammary gland	Fibrocystic Disease	[1]
**MCF-7**	Epithelial cell	Epithelial	Breast; Mammary gland	Adenocarcinoma	[1]
**MDA-MB-157**	Epithelial cell	Epithelial	Breast; Mammary gland; Medulla	Carcinoma; Medulallary	[1]
**MDA-MB-231**	Epithelial cell	Epithelial	Breast; Mammary gland	Adenocarcinoma	[1]
**MDA-MB-435**	Epithelial cell	Epithelial	Breast; Mammary gland	Carcinoma; Metastatic	[1]
**MDA-MB435-S**	Melanocyte	Spindle-shaped	Breast; Mammary gland	Melanoma	[1]
**NCI-H226**	Epithelial cell	Epithelial	Lung	Squamous Cell Carcinoma; Mesothelioma	[1]
**OVCAR-3**	Epithelial cell	Epithelial	Ovary	Adenocarcinoma	[1]
**T-47D**	Epithelial cell	Epithelial	Breast; Mammary gland	Carcinoma; Ductal	[1]
**HEMC**	Epithelial cell	Cuboidal with a characteristic pattern of swirled cells; refractile	Breast	Non-applicable	[1]

Non-applicable means that the cell line comes from healthy tissue isolation.

References

1. American Type Culture Collection 2024. The global bioresource center. Availabe online: https://www.atcc.org/
2. Briem, E.; Ingthorsson, S.; Traustadottir, G.A.; Hilmarsdottir, B.; Gudjonsson, T. Application of the D492 Cell Lines to Explore Breast Morphogenesis, EMT and Cancer Progression in 3D Culture. *J Mammary Gland Biol Neoplasia* **2019**, *24*, 139-147, doi:10.1007/s10911-018-09424-w.
